# Comparison of autonomic reactivity to trauma and nightmare imagery: A Pilot Study

**DOI:** 10.1093/sleepadvances/zpae060

**Published:** 2024-08-13

**Authors:** Christopher M McGrory, Augustus Kram Mendelsohn, Suzanne L Pineles, Natasha B Lasko, Vladimir Ivkovic, Mabelle Moon, Doga Cetinkaya, Oren Bazer, Elizabeth Fortier, Anne Kelly, Laura B Bragdon, Kimberly A Arditte Hall, Kaloyan Tanev, Scott P Orr, Edward F Pace-Schott

**Affiliations:** National Center for PTSD, Women’s Health Sciences Division at VA Boston Healthcare System, USA; Department of Psychiatry, Boston University Chobanian & Avedisian School of Medicine, USA; Department of Psychiatry, Massachusetts General Hospital, USA; National Center for PTSD, Women’s Health Sciences Division at VA Boston Healthcare System, USA; Department of Psychiatry, Boston University Chobanian & Avedisian School of Medicine, USA; Department of Psychiatry, Massachusetts General Hospital, USA; Department of Psychiatry, Harvard Medical School, USA; Department of Psychiatry, Massachusetts General Hospital, USA; Department of Psychiatry, Harvard Medical School, USA; Department of Psychiatry, Massachusetts General Hospital, USA; Department of Psychiatry, Massachusetts General Hospital, USA; Department of Psychiatry, Massachusetts General Hospital, USA; Department of Psychiatry, Massachusetts General Hospital, USA; Department of Psychiatry, Massachusetts General Hospital, USA; Department of Psychiatry, Grossman School of Medicine, New York University, USA; Department of Psychology and Philosophy, Framingham State University, USA; Department of Psychiatry, Massachusetts General Hospital, USA; Department of Psychiatry, Harvard Medical School, USA; Department of Psychiatry, Massachusetts General Hospital, USA; Department of Psychiatry, Harvard Medical School, USA; Department of Psychiatry, Massachusetts General Hospital, USA; Department of Psychiatry, Harvard Medical School, USA

**Keywords:** PTSD, psychophysiology, sleep, exposure therapy

## Abstract

**Study Objectives:**

Trauma-related nightmares (TRNs) are a hallmark symptom of PTSD and are highly correlated with PTSD severity and poor sleep quality. Given the salience and arousal associated with TRNs, they might be an effective target for imaginal exposures during Prolonged Exposure (PE) therapy. As a first step in this line of research, the current study compared participants’ emotional reactivity during recollection of TRNs to their recollection of the index traumatic event.

**Methods:**

Seventeen trauma-exposed participants with clinical or sub-clinical PTSD who reported frequent TRNs engaged in script-driven imagery using scripts depicting their index trauma and their most trauma-like TRN. Heart rate (HRR), skin conductance (SCR), corrugator EMG (EMGR) responses, and emotional ratings were recorded.

**Results:**

HRR, SCR, and EMGR did not differ significantly between trauma-related and TRN scripts. Bayesian analyses confirmed support for the null hypothesis, indicating no differences. With the exception of “Sadness,” for which TRNs elicited significantly lower ratings than trauma scripts, individual emotion ratings showed no significant differences, suggesting likely parity between the emotionality of trauma-related and TRN recollections.

**Conclusions:**

Together, TRN content elicited psychophysiological reactivity similar to that of the index trauma in this pilot study. Upon replication, studies testing TRNs as potential targets for imaginal exposures during PE may be warranted.

**Statement of Significance**

While Prolonged Exposure (PE) therapy can be an effective trauma-focused therapy, room for improvement exists. Repeatedly recounting details of a recent trauma-related nightmare could serve as one such approach, providing emotionally relevant targets for PE therapy. Given the fact that physiological arousal during PE has been shown to facilitate extinction learning, this investigation aimed to compare the psychophysiological and emotional reactivity while recollecting nightmares related to a traumatic experience and the original trauma. Results showed reactivity largely parallels one another. Findings suggest nightmare content might be a potential target for trauma-focused therapy.

## A contribution to the Festschrift issue of *SLEEP Advances* honoring Robert Stickgold, PhD

Robert Stickgold’s long-term and passionate interest in dream science has led to a prolific record of publications on the cognitive and clinical neuroscience of dreaming. His work includes pioneering analyses of the formal aspects of dream phenomenology and cognitive neuroscience including a cover article in *Science* [1]. Regarding Trauma-related nightmares (TRNs), he authored an early study of 9/11-related dreams as well as two influential papers on the potential mechanism of eye movement desensitization and reprocessing therapy that were informed by REM-sleep physiology and dream research [2–4]. He has been instrumental in bringing sleep and dream science to the general public through lectures, print and electronic media, and a recent widely read and reviewed book [5]. Most recently he has been developing a novel technique of targeted dream incubation (TDI) that focuses on a specific topic during sleep onset and has been shown to subsequently promote more creative thought about this topic following sleep [6, 7]. He is currently working with his colleague Adam Horowitz and an American/Israeli software company, to develop a protocol to use TDI for the treatment of PTSD, which will initially focus on survivors of the October 7th Hamas attacks in Israel, but eventually much more widely, including survivors in Gaza. The idea is to use TDI to help people dream about traumatic events they have experienced in a way that will “unfreeze” the normal block that prevents people with PTSD from dreaming about the trauma in ways that will help them heal. Bob has been a true pioneer in investigating the mechanisms of normal dreams and nightmares, and applying dream science for the benefit of those suffering posttraumatic nightmares.

## Nightmares

TRNs with content resembling an experienced traumatic event have been characterized as a highly specific “hallmark” symptom of PTSD [8, 9]. Nightmares (NMs) are experienced by up to 70% of individuals with PTSD and more so in those with comorbid disorders [10, 11]. Nightmare frequency, distress, and replicative quality are noteworthy characteristics that indicate more severe and persistent PTSD [12–14]. TRNs are often highly distressing [15, 16] and associated with more severe insomnia [10] and poorer overall sleep quality [17, 18]. Additionally, NMs are an example of the bidirectional nature of sleep impairment and PTSD; NMs are exacerbated by PTSD and are a risk factor for the development of PTSD [19, 20].

TRNs disrupt sleep by producing anticipatory fear and anxiety surrounding sleep, resulting in difficulty initiating sleep, sleep avoidance, or behaviors not conducive to sleep (e.g. leaving the lights on) [21, 22]. They can cause awakenings from sleep and acute physiological arousal that can make returning to sleep difficult [18, 21] and compromise sleep quality, which may impair consolidation of fear-extinction memories [23, 24]. NMs also may be a risk factor for PTSD; for example, veterans who reported having NMs had higher PTSD severity at both post-deployment baseline and at 6-month follow-up, at which time 41% of NM sufferers continued to meet criteria for PTSD, compared to <10% of those who did not experience NMs [13]. Sleep disturbances tend to be resistant to standard psychological and pharmaceutical interventions [25, 26] and contribute to poor PTSD treatment outcomes [27, 28].

### Prolonged Exposure therapy

Prolonged Exposure (PE) therapy is a first-line treatment for PTSD. PE involves repeatedly recounting the details of the index trauma (i.e. the event causing PTSD symptoms) with a therapist during imaginal exposures and while confronting actual situations that are avoided during in vivo exposure exercises between therapy sessions [29]. While PE can produce significant reductions in PTSD symptoms [30], there is considerable room for improvement. Notably, high dropout rates (e.g. 55.8% [31]) prevent treatment-seeking individuals from fully benefiting from PE and most still meet diagnostic criteria for PTSD after completion of PE [32, 33]. Nighttime symptoms such as NMs often do not change in response to PE treatment of traumatic memories and, when they do, PTSD symptoms frequently remain in clinical ranges [34, 35].

The neurocognitive underpinnings of PE involve fear-extinction learning. Extinction diminishes the fear response associated with traumatic memories by forming inhibitory memories (extinction memories) that counter the fearful memories. It is known that increased physiological arousal during PE can facilitate extinction learning [36, 37]. The relationship between increased physiological arousal, evidenced by increased heart rate (HR), skin conductance (SC), and facial electromyography, and improved fear-extinction is documented in both clinical and experimental paradigms and understood to be due to improved acquisition, consolidation, and retrieval of inhibitory learning [37–39]. The development of methods that increase physiological arousal within PE sessions is a possible way to improve exposure treatments [40, 41]. Given that the traumatic event is temporally distant, compared to TRNs, the memory of the latter may be more salient and detailed than trauma memories, per se. Similarly, it is important to note that PE requires a sufficiently detailed memory, which can be compromised due to trauma-related events (e.g. head injury, blackout, etc.). As such, repeatedly recounting details of a recent TRN could provide emotionally relevant targets for PE therapy that may be better tolerated during initial treatment sessions or be more accessible in the case of compromised trauma memory. The present study investigated whether recollecting a TRN, using a standardized script-driven imagery (SDI) paradigm previously developed by our group (e.g. [42, 43]), could produce a substantive emotional response as indexed by heightened psychophysiological reactivity.

### Script-driven imagery

SDI involves listening to and imagining brief audio-recorded vignettes that recount an individual’s personal traumatic experiences as well as other standard neutral and emotional scenes. During SDI, heart rate (HRR), skin conductance (SCR), and facial electromyographic (e.g. corrugator supercilii EMGR) responses are recorded along with subjective ratings of emotional experiences. Together, the physiological measures have been shown to serve as an objective biomarker [44] and measure of PTSD symptom severity, and are capable of distinguishing those with versus without clinician-diagnosed PTSD (for review see [38],). Further exemplifying the mechanisms of fear-extinction and PE, heightened physiological reactivity to trauma scripts at pretreatment has been found to be associated with better PTSD treatment outcomes [45].

TRNs are rarely a completely veridical replay of traumatic experiences; however, the intense emotional response they can generate makes them a potential target for the SDI procedure [46]. Research using the SDI procedure has shown that psychophysiological reactivity is driven by the emotional significance of a memory irrespective of whether it took place in subjective or objective reality. For example, psychophysiological reactivity to scripts recounting recollections of personal, but highly unlikely, events (e.g. purported traumatic encounters with space aliens) is comparable to that produced by traumatic events that took place in the physical world [47]. The SDI procedure previously has been used to assess reactivity to TRN scripts in the context of a treatment study [48, 49]. Exposure, Relaxation, and Rescripting Therapy (ERRT) is an evidence-based treatment for PTSD-related NMs that involves targeting the worst TRNs through exposure exercises and rescripting of the dream content. Using SDI, Rhudy et al. [49, 50] showed that TRN scripts elicited significantly more reactivity than neutral scripts and, subsequently, that ERRT treatment reduced both physiological and subjective reactivity to TRN scripts. Davis, et al. [48] found that decreased HRR and SCR to TRN scripts after receiving ERRT were associated with improved psychological outcomes. However, these previous studies did not compare reactivity to TRNs and trauma memories—an important comparison for establishing the feasibility and potential utility of using TRNs in the context of PE.

The present study used SDI to compare psychophysiological and subjective reactivity to TRN scripts and trauma-memory scripts. The goal was to assess whether recollection of TRNs produced comparable or greater emotional reactivity as compared to recalling the traumatic event to which the TRN was related. Given the salience and greater temporal proximity of TRNs, we hypothesized that HRR, SCR, and corrugator EMGR and emotional ratings associated with the TRN, would equal or exceed the responses produced while recollecting the traumatic event itself.

## Methods

### Participants

Individuals reporting TRNs were recruited from the community through social media and websites dedicated to research participation. Forty participants consented to begin the study, 25 individuals completed at least some portion of the protocol described below, and 17 completed all portions of the study (completers’ mean age = 27.47 years, SD = 10.33, range 19-54 years). The majority of participants were self-identified female (88.2%), white (75%), and earning under $50 000 per year (58.82%). Inclusion criteria were having experienced a DSM-5 PTSD criterion-A event and reporting at least 2 NMs per week with some of the NM content directly related to the criterion-A event, and meeting criteria for a DSM-5 PTSD diagnosis or partial PTSD (endorsing the requisite number of symptoms in at least three of the four diagnostic clusters). Exclusion criteria included lifetime history of psychotic, bipolar, autism spectrum or other neurodevelopmental disorders, current active suicide risk, current substance-use disorder or urine toxicology positive for drugs of abuse, potentially confounding neurologic or other serious medical conditions, history of sleep apnea, and current regular use of benzodiazepines or prazosin. The study protocol was approved by the Partners Healthcare Institutional Review Board. Participants provided written informed consent and were paid for their participation.

### Procedures

Following telephone screening, participants completed an in-person consent and pre-interview visit during which they wrote an account of their trauma (or the trauma most closely related to their NMs for participants who had experienced more than one trauma) and completed a checklist of any bodily sensations they may have experienced during the event [43, 51]. They then completed the Life Events Checklist (LEC [52]) and the PTSD Checklist for DSM-5 (PCL-5 [53]) focusing on the event to which their nightmares were related, and a urine toxicology screening. Participants then completed a clinical interview during which the Structured Clinical Interview for the DSM-5 (SCID-5 [54]), the Clinician-Administered PTSD Scale for DSM-5 (CAPS-5 [55]), and the Clinical Interview for DSM-5 Sleep Disorders Module (SCISD [56]) were administered.

**Figure 1.**
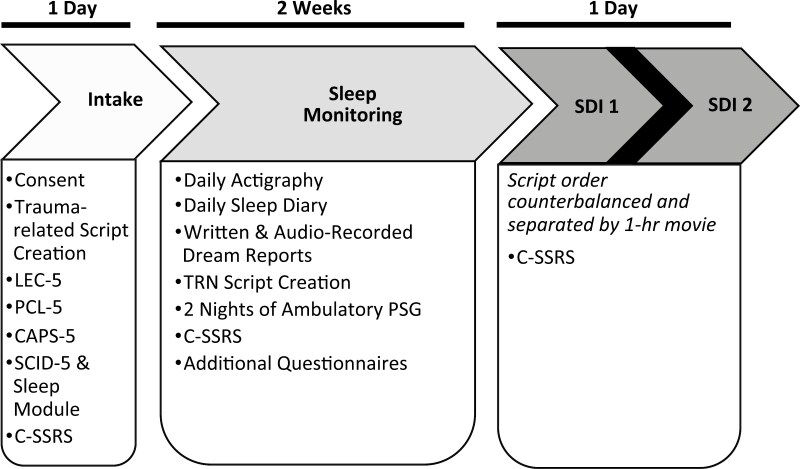
Overview of procedures. CAPS-5, Clinician-Administered PTSD Scale for DSM-5; C-SSRS, Columbia Suicide Severity Rating Scale; LEC-5, Life Events Checklist for DSM-5; PCL-5, PTSD Checklist for DSM-5; PSG, polysomnography; SCID-5, Structured Clinical Interview for DSM-5; SDI, script-driven imagery; TRN, Trauma-related nightmare.

Participants who met study inclusion criteria completed 14 days of sleep monitoring with the Actiwatch 2 (Philips Respironics, Bend, OR; for details see supplementary material in [57]) and a sleep diary (Evening–Morning Sleep Questionnaire [58, 59]) that included a nightmare questionnaire [46]. During this period, using a time-stamping audio recorder, they recorded dream reports and a checklist of bodily sensations they experienced when awakened by an NM or that they recalled having occurred during the night upon awakening. Each participant also completed at least 2 nights of ambulatory polysomnography (PSG) using the Somte-PSG monitor (Compumedics USA, Inc., Charlotte, NC). The first night was an acclimation/diagnostic night and the second night provided the PSG data to be analyzed (for more details see [60, 61],) that will be presented in a subsequent report. Participants also completed online self-report questionnaires on sleep quality, chronotype, anxiety and depressive symptoms, trauma history, and personality traits (see below). Following the 14-day sleep-monitoring period, participants completed both SDI sessions in 1 day (Figure 1). The Columbia Suicide Severity Rating Scale (C-SSRS) was administered at every in-person (consenting, evenings with PSG, SDI appointments) and virtual visit (screening interview) [62].

#### Script-driven imagery.

Both the trauma narrative written at intake and audio-recorded NM reports from the 14-day sleep-monitoring period were transcribed and used to create personalized audio-recorded scripts for use during the SDI sessions. Two non-overlapping scripts describing portions of the trauma and NM reports were written, resulting in four personalized scripts in total. The recorded NM selected for the TRN scripts was the one most closely resembling the trauma report the participant wrote at intake, as determined by the senior investigators based on the most relevant trauma-related details. Scripts varied in length from four to eight sentences, did not exceed 42 seconds in length, were written in the second-person present tense, and were recorded by a male experimenter. Both trauma and TRN scripts were created using as many as possible grounding characteristics (e.g. location, date, time) and bodily sensations that participants reported accompanied the traumatic event or TRN, respectively. Analyses comparing the linguistic features of the trauma and TRN scripts revealed that the trauma and TRN scripts did not differ linguistically (i.e. number of positive, negative, and somatosensory words) but participants did endorse more physical symptoms when recounting the trauma memory [63].

Each participant’s two SDI sessions were conducted in the early to mid-afternoon. Each session included three standard neutral scripts and two different personal scripts containing material from either the participant’s original index trauma report (Trauma script) or the selected TRN (TRN script), resulting in a total of five scripts for each SDI session. Neutral scripts included scenes of sitting in a lawn chair, looking out at trees, and laying on the beach and did not differ between SDI sessions. The two SDI sessions were separated by a 1-hour break during which all participants watched the same neutral movie entitled *Our Planet: Coastal Seas* [64]. The order of Trauma vs. TRN script presentation was counterbalanced across participants.

SDI procedures were adapted from standardized protocols [43, 51] and began by instrumenting participants with physiological recording equipment and headphones while seated, a procedure lasting approximately 15 minutes. The experimenter then left the room while participants sat quietly for a 5-minute baseline recording period before listening to a 3-minute relaxation script. SC, HR, and corrugator supercilii electromyogram (EMG) levels were recorded during SDI procedures using the Biopac MP-150 system (described below). Participants were asked to remain seated and still throughout the procedures.

**Figure 2.**
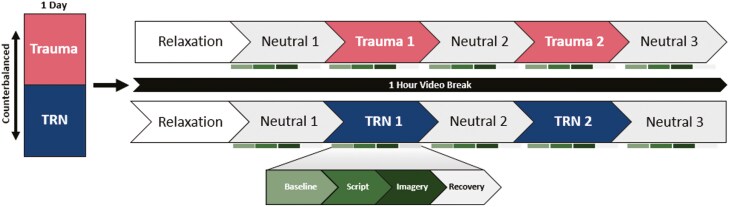
Script-driven imagery procedure overview. Participants were counterbalanced to complete the SDI procedure using the Trauma scripts either before or after the SDI procedure using the TRN scripts. TRN, trauma-related nightmare.

Each script included baseline, listening, imagery, and recovery periods (approximately 30 seconds each) followed by the completion of eleven 12-point rating scales that assessed subjective experiences during the script (Figure 2). Immediately after listening to the script, participants imagined the scene as vividly as possible, as if they were in the situation until a tone signaled the beginning of the recovery period during which participants were instructed to stop imagining and relax. At the end of the recovery period, the participant made their eleven subjective ratings of the imagery experience. The next script was presented at least 60 seconds after completion of the subjective reports and when the 5-second HR average value had consistently returned to within 5% of the previous 30-second average HR level during baseline and other physiological measures had stabilized or after 180 sec had elapsed, whichever came first. This was accomplished using the Biopac AcqKnowledge 4.1.5 software (Biopac Systems Inc., Goleta, CA) output, which was programmed to show real-time, 5-sec average, and 30-second average HR. Experimenters who administered the SDI were trained by the developer of the procedures and instructed to not advance to the next script until they were certain autonomic levels had stabilized or 180 seconds had elapsed.

#### Physiological equipment and recording.

Physiological biosignals were collected using the Biopac (Biopac Systems Inc.) MP150 system with AcqKnowledge 4.1.5 software and ECG100C, EMG100C, and EDA100C transducer modules for recording HR, EMG, and SC levels, respectively. The physiological biosignals were sampled at 2000 Hz for HR and EMG, and 31.25 Hz for SC. SC was measured using two 11 mm Ag/AgCl disposable electrodes (Biopac EL507) that were filled with isotonic paste (Biopac Gel 101) and placed on the hypothenar surface of the participant’s nondominant hand separated by 14 mm. The skin above the right corrugator supercilii was prepared with a wet abrasive pad, cleaned with an alcohol wipe, and allowed to dry before placing two BIOPAC EL254S Ag-AgCl 4-mm TP shielded recording electrodes filled with CG04 Saline Base Signa Gel. EMG was filtered with a 90 Hz high-pass filter, rectified, and integrated over a 250 milliseconds time constant before analysis. ECG was recorded using EL503 Ag/AgCl 11 mm electrodes filled with CG04 Saline Base Signa Gel placed below the right clavicle in the second intercostal space and on the left eighth intercostal space after cleaning the skin with alcohol.

#### Assessments completed online.

The following self-report assessments were completed online by participants during the 14-day sleep recording period using the Research Electronic Data Capture (REDCap^TM^) system (© 2013, Vanderbilt U). **The Insomnia Severity Index** (ISI): the ISI is a 7-item self-report measure of insomnia disorder symptomology (Bastien et. al. 2001). The **Pittsburgh Sleep Quality Index** (PSQI): the PSQI is the most widely used self-report assessment of sleep quality and consists of 19 items encompassing 7 sleep-quality factors (Buysse et al., 1989). The **PSQI PTSD Addendum** (PSQI-A): this addendum queries the presence and frequency of seven disruptive nocturnal behaviors believed to represent PTSD-specific sleep disturbances (Germain et al., 2005). **Epworth Sleepiness Scale** (ESS): the ESS is the standard assessment of excessive daytime sleepiness (Johns, 1991). **Morningness–Eveningness Questionnaire** (MEQ): the MEQ is the oldest and most commonly used measure of chronotype (Horne & Ostberg, 1976). **Spielberger State-Trait Anxiety Inventory, Trait** (STAI-T): the STAI-T is a standardized and validated measure of trait anxiety [65]. The **Quick Inventory of Depressive Symptomatology** (QIDS): the QIDS is a 16-item measure of depressive symptom severity that covers 9 criterion areas of depression [66].

#### Outcome variables.

To calculate physiological reactivity scores (i.e. HRR, SCR, and EMGR) for each script, mean HR, SC, and EMG levels during the baseline period were subtracted from their respective imagery period mean levels. These change scores were square root transformed to reduce heteroscedasticity; negative signs were retained after calculating the square root of absolute values to capture the direction of change for responsivity to neutral scripts. The 11 subjective rating scores also served as outcome variables.

### Statistical analyses

Responses to the Trauma and TRN scripts were initially analyzed using mixed ANOVA that included one between-participant factor, Order (Trauma SDI first, TRN SDI first), and two within-participant factors, Condition (TRN vs. Trauma script) and Valence (Trauma/TRN vs. Neutral) in RStudio Version 2022.12.0 + 353. For HRR, SCR, and EMGR values, outliers (i.e. values greater than 3 SD from the mean) were replaced with their value at 3 SD. The number of outliers was small with 2 HRR outliers, 1 SCR outlier, and 2 EMGR outliers replaced. Pairwise tests were conducted to decompose significant interactions. Shapiro–Wilk tests were used to test distributions for normality. For non-normal distributions, analyses were repeated using non-parametric Wilcoxon signed-rank tests. Separate Wilcoxon signed-rank tests were used for the Valence factor in the Trauma Condition (Trauma vs. Neutral), the Valence factor in the TRN Condition (TRN vs. Neutral), and the Condition factor within the negative-Valence stimuli (Trauma vs. TRN). In all cases, results using non-parametric analyses were consistent with those using ANOVA. Given the initial results in favor of the null hypothesis for the majority of analyses, Bayesian repeated measures ANOVAs with default priors were then conducted using JASP 0.18.1 (JASP Team, 2023). Bayes factors (BF) are a useful approach as they allow for more confidence in the null results compared to traditional null hypothesis testing [67]. When comparing the alternate hypothesis (H_1_) to the null hypothesis (H_0_), the Bayes Factor is written BF_10_. All post hoc analyses were repeated using Bayesian approaches in JASP.

## Results

### Sample psychological and sleep characteristics

As shown in Table 1, 10 (58.8%) participants met full diagnostic criteria for PTSD on the CAPS-5 assessment, all others met subthreshold criteria (i.e. three of four diagnostic criteria in addition to criterion-A were met). Additionally, on average, participants reported clinically significant sleep disturbances on the ISI and PSQI, as well as elevated anxiety on the STAI-T and a moderate level of depressive symptoms (Table 1). Sleep characteristics measured objectively with the Actiwatch 2 and subjectively with a daily sleep diary can be found in Table 1; ambulatory PSG results will be reported in future manuscripts.

**Table 1.** Mean and SE for Psychological Measures and Actigraphy Sleep Variables

Variable	*n*	Mean	SE	Minimum	Maximum
Clinical measures					
Full PTSD diagnosis (CAPS-5)	10/17				
CAPS-5	17	28.06	2.94	5	47
PCL-5	17	46.29	3.17	21	63
ISI	16	12.13	1.42	3	21
PSQI	16	9.31	1.12	3	17
PSQI-A	16	12.13	1.20	1	20
ESS	16	6.94	1.02	0	13
MEQ	16	48.13	2.52	24	62
STAI-T	16	48.63	3.06	27	72
QIDS	16	14.81	1.17	5	23
Actiwatch					
TST (minutes)	12	380.12	23.45	251.08	509.62
Sleep efficiency (%)	12	83.49	1.61	69.43	96.34
Sleep diary					
TST (minutes)	13	414.48	21.29	297.92	528.44
Sleep efficiency (%)	13	88.35	2.80	77.25	94.51

CAPS-5, Clinician-Administered PTSD Scale for DSM-5; ESS, Epworth Sleepiness Scale; ISI, Insomnia Severity Index; MEQ, Morningness–Eveningness questionnaire; PCL-5, PTSD Checklist for DSM-5; PSQI, Pittsburgh Sleep Quality Index; PSQI-A, PSQI addendum for PTSD; PTSD, posttraumatic stress disorder; QIDS, Quick Inventory of Depressive Symptomatology; STAI-T, Spielberger State-Trait Anxiety Inventory, Trait; TST, total sleep time.

### Physiological reactivity to trauma, TRN, and neutral scripts

**Figure 3.**
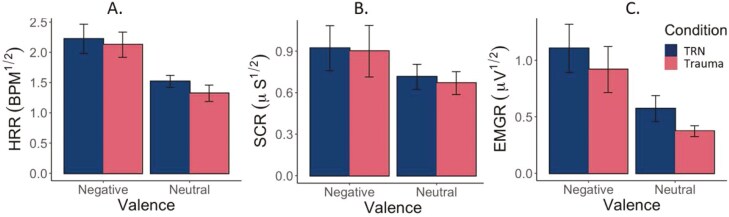
Mean (and SE) heart rate, skin conductance, and corrugator EMG responses for standard neutral, TRN, and trauma-related imagery. Results show no significant differences between reactivity to TRN and Trauma scripts for HRR, SCR, or EMGR during SDI procedures. Error bars are SE. Square-root transformed values reported on the *y*-axis. Untransformed HRR change values are as follows: TRN-Negative HRR = 4.52 BPM, Trauma-Negative HRR = 5.08 BPM, TRN-Neutral HRR = 2.31 BPM, Trauma-Neutral HRR = 1.75 BPM. Comparison of untransformed data yielded the same results as transformed data. BPM, beats per minute; µS, microsiemens; µV, microvolts; HRR, heart rate response; EMGR, corrugator electromyogram response; SCR, skin conductance response; TRN, Trauma-related nightmare.

Participants demonstrated significantly greater HRR and EMGR to negative (Trauma or TRN scripts), compared to the standard neutral scripts (HRR: F(1,16) = 14.66, *p* = .001, *η*_*p*_^2^ = 0.48; EMGR: F(1,16) = 14.40, *p* = .002, *η*_*p*_^2^ = 0.20). There was also a statistical trend supporting greater SCR to negative (Trauma or TRN scripts), compared to the standard neutral scripts (F(1,16) = 3.92, *p* = .065, *η*_*p*_^2^ = 0.20; Figure 3). In contrast, HRR, EMGR, and SCR did not differ between Trauma and TRN scripts (Figure 3). Neither the Condition (Trauma vs. TRN) main effect nor the Condition × Valence interactions reached significance (all *p*s > .23).

Findings were further supported using Bayesian analyses. For interpretation of Bayesian results, (including the designations “anecdotal,” “substantial,” and ‘strong’) please refer to the Table 2 legend. Strong evidence was found in support of participants demonstrating greater HRR and EMGR to negative (Trauma or TRN scripts), compared to the standard neutral scripts (Table 2; Figure 3) and there was also anecdotal evidence supporting greater SCR to negative (Trauma or TRN scripts), compared to the standard neutral scripts (Table 2; Figure 3). Anecdotal evidence for the null hypothesis was found for the Condition (Trauma vs. TRN) main effects and there was substantial evidence supporting no difference between Trauma and TRN imagery for HRR, SCR, or EMGR in pairwise analyses (BF_10_ = 0.32, 0.26, and 0.26, respectively; Figure 3). Additionally, for the Condition × Valence interactions, there was no evidence for the alternative hypothesis with the Bayesian repeated measures ANOVA (BF_10_ = 0.32–0.39).

**Table 2.** Bayes Factors (BF_10_) Obtained for Valence, Condition, and their Interaction

Measure	Valence (negative vs. neutral)	Condition (trauma vs. TRN)	Valence X condition
HRR	27.90^*^	0.45	0.34
SCR	1.42	0.40	0.52
EMGR	25.70^*^	0.70	0.72

BF_10_ values greater than 1 provide anecdotal (1–3), substantial (3–10), strong (10–30), very strong (30–100), and decisive (>100) support for the alternative hypothesis; BF_10_ values <1 provide anecdotal (1–0.33), substantial (0.33–0.10), strong (0.10–0.03), very strong (03–0.01), and decisive (<0.01) support for the null hypothesis [67]. HRR, heart rate response; EMGR, corrugator electromyogram response; SCR, skin conductance response; TRN, Trauma-related nightmare.

^*^strong evidence for the alternative hypothesis.

**Figure 4.**
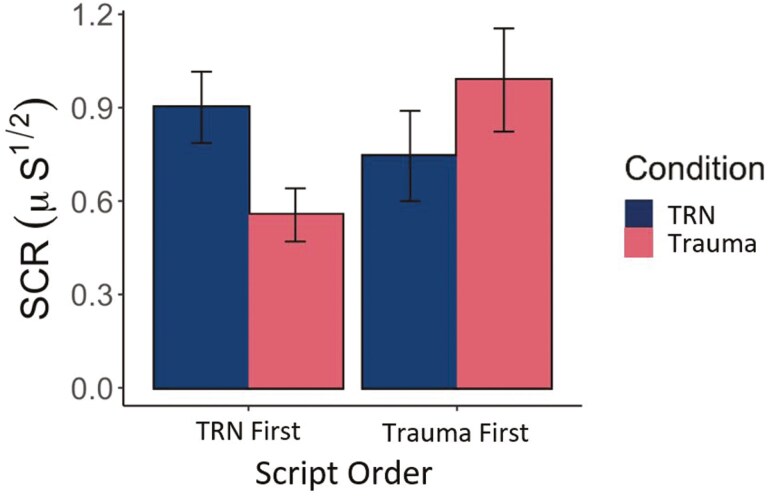
SCR mean and SE for individuals imagining trauma first or TRN first during the first SDI session. Results show that the Trauma or TRN scripts presented first produced larger SCR. Error bars are SE. SDI, script-driven imagery; µS, microsiemens; SCR, skin conductance response; TRN, Trauma-related nightmare.

When Order (whether the Trauma or TRN scripts were presented first during the first SDI session) was added to the models, no Order main effect or Condition × Order interaction was found for HRR (F(1,15) = 0.01, *p* = .925, *η*_*p*_^2^ < 0.001) or EMGR (F(1,15) = 0.24, *p* = .635, *η*_*p*_^2^ = 0.02). This was supported by Bayesian analyses with anecdotal evidence in support of the null hypotheses for the Order main effects (BF_10_ = 0.46–0.60) and Condition × Order interactions (BF_10_ = 0.47–0.52). However, for SCR there was a significant Condition × Order interaction (F(1,15) = 13.26, *p* = .002, *η*_*p*_^2^ = 0.47), such that SCR values were higher on the first SDI session compared to the second, irrespective of the condition counterbalanced to be presented first (Trauma or TRN; *t*(15) = −2.93, *p* = .045; Figure 4). Similarly, the Bayesian analyses provided strong evidence for this interaction (BF_10_ = 11.48) and pairwise comparison for TRN First (BF_10_ = 15.48) (Figure 4).

### Subjective reactivity to trauma and TRN scripts

As in the case of physiological responses, there was significant and decisive support for a difference (*p* < .0001; BF_10_ > 1000) between standard neutral versus negative (i.e. Trauma or TRN) scripts for individually rated emotions with directionality determined by the valence of the rated emotion. The individually rated emotions showed neither significant differences between Trauma and TRN imagery (Table 2) nor Condition × Valence interactions with the exception of ‘Sadness’ (F(1,16) = 6.84, *p* = .02, *η*_*p*_^2^ = 0.30) which was rated higher in the Trauma condition. When a Bonferroni correction for multiple comparisons (critical alpha 0.0045) was applied, this effect was no longer significant. The Bayesian approach largely verified these findings as there was anecdotal evidence supporting a lack of difference between Trauma and TRN for the majority of emotion ratings (Table 3) and evidence for a Condition × Valence interaction only for the “Sadness” rating (BF_10_ = 7.15). However, the Bayesian analyses also yielded anecdotal-level evidence suggesting a difference in “Pleasantness” and “Happiness,” in addition to “Sadness,” between Trauma and TRN scripts such that the Trauma scripts were rated with less pleasantness, less happiness, and more sadness, than the TRN scripts (Table 3).

**Table 3.** Mean and SE Self-reported Ratings to TRN and Trauma-Related Imagery

	TRN scripts		Trauma-related script					
Rating	Mean	SE	Mean	SE	t(16)	*p*	*Cohen’s d*	B_10_
Pleasantness	1.00	0.37	0.06	0.04	−2.61	.081	0.86	2.32
Arousal	9.38	0.51	9.44	0.62	0.17	.998	−0.03	0.32^†^
Control	2.21	0.54	1.62	0.51	−1.07	.71	0.27	0.37
Vividness	9.74	0.44	10.06	0.47	0.62	.924	−0.17	0.37
Happiness	0.62	0.32	0.09	0.06	−1.77	.321	0.55	1.35
Sadness	6.29	0.88	9.32	0.67	3.28^*^	.022	−0.94	6.49**
Anger	7.44	0.87	9.12	0.49	1.88	.274	−0.57	0.91
Fear	9.71	0.54	10.03	0.53	0.56	.937	−0.15	0.35
Disgust	6.79	0.86	7.59	0.92	0.87	.821	−0.22	0.39
Surprise	5.41	0.87	5.71	0.92	0.32	.989	−0.08	0.32^†^
Guilt	5.09	1.01	6.47	0.87	1.88	.276	−0.36	0.70

All ratings are subjective reports following imagery on a 0–12 scale (e.g. not at all happy to completely happy). TRN, trauma-related nightmare. *p*-values represent within-participant differences in means between response to TRN and Trauma scripts.

^*^ *p < *.05. **substantial evidence for the alternative hypothesis.

^†^substantial evidence for the null hypothesis.

## Discussion

The aim of this study was to compare psychophysiological and subjective reactivity during recollections of trauma-related nightmare (TRN) and actual trauma (Trauma) experiences using the SDI procedure. We hypothesized that reactivity to TRNs, as indexed by HR, SC, and corrugator EMG responses, as well as by subjective emotion ratings, would equal or exceed the responses produced while imagining the traumatic event. Results showed anecdotal support for there being no difference between the magnitude of psychophysiological reactivity during imagery of trauma memory and imagery of a TRN when measured by HR and corrugator EMG responses. Similar results were held for SC responses with the exception that, when separated by a short (1-hour) interval, SCR was greater during imagery of the Trauma or TRN scripts when presented during the first, compared to the second, SDI session. This may reflect a tendency for SC to habituate over repeated presentations of similar, but not necessarily identical, stimuli [68]. For subjective ratings, there was no support for differences between the majority of emotional responses to Trauma and TRN scripts, with the exception of Pleasantness, Happiness, and Sadness such that TRN scripts were rated less negatively. Support for these differences should be interpreted cautiously given the fact that the significant difference in Sadness ratings was lost when Bonferroni correction was applied, and the results using Bayesian analyses only supported differences for ratings of Sadness, Pleasantness, and Happiness at the anecdotal level. With that said, these findings replicate and extend the work of Rhudy and colleagues [50] demonstrating greater HRR, SCR, and EMGR, and negative ratings for TRN imagery, compared to neutral imagery.

In considering the clinical utility of having TRN memories available to use in PE sessions in addition to the traditionally used trauma memories, there are several possible uses: First, TRN memories provide similar emotional salience as trauma memories that could be titrated in intensity according to need. That is, if the patient is excessively fearful and avoidant of their trauma memory, the TRN could serve as initial exposure targets to ease them into PE. Second, if the trauma memory is inaccessible, distorted, or over-rehearsed, the TRN could serve as the focus memory during imaginal exposure or be used as a supplemental exposure target to boost emotional intensity during imaginal exposure sessions to strengthen extinction learning. Inhibitory learning is believed to require a degree of physiological arousal in order to generate robust extinction memories. As such, reducing arousal to fearful memories by intentionally or incidentally presenting de-arousing safety signals can impede extinction learning during exposure [36, 37, 69]. Adequately powered randomized clinical trials must ultimately demonstrate non-inferiority to traditional PE or if the inclusion of TRNs in treatment can provide added clinical benefit.

Several limitations pertain to this study. First, our investigation was conducted during the height of the COVID-19 pandemic when the safety of, and enthusiasm for, in-person research was severely compromised. Despite our best efforts to effectively recruit participants, the small sample size must be acknowledged; findings should be considered preliminary. Relatedly, participants were not explicitly asked to refrain from the use of caffeine, alcohol, food, exercise, or other factors which might impact autonomic nervous system functioning on the day of testing. However, this limitation is mitigated by the use of a resting baseline, an initial relaxation script, and the focus on change scores comparing differences in physiological response to the imagery as compared to baseline periods rather than absolute HR, SC, and EMG levels which would be more susceptible to inter-individual differences and artifact. Second, females and those self-identifying as White were overrepresented in the current sample, potentially limiting generalizability [70, 71]. Third, not all participants met the full diagnostic criteria for PTSD. However, physiological reactivity in sub-threshold PTSD has been shown to mirror that of full PTSD when elicited in a fear conditioning paradigm, which supports the generalizability of the current findings [72]. With respect to methodology, we were initially concerned that the 1-hour break between administration of the trauma and TRN scripts would be insufficient. However, the absence of an interaction effect between the order of scripts presented and reactivity (except for SCR) shows the robustness of responses to both the Trauma and TRN scripts. Additionally, the duration since trauma was not specifically collected and therefore, the theoretical benefits of temporal proximity remain untested here. However, a number of previous publications have included time since trauma as a covariate and did not find significant associations with any outcome measures [73, 74]. Lastly, given the differences between SDI and the verbal retelling of experiences during imaginal exposures in Prolonged Exposure therapy, any clinical implications are theoretical.

## Conclusion

The present study contributes to our understanding of the psychophysiological underpinnings of traumatic nightmares and supports the potential utility of directly targeting TRNs in trauma-focused treatments. The apparent equivalence of psychophysiological reactivity to TRNs and trauma memories supports the possibility of using TRN imagery, on an occasional, situational, or repeated basis, as an additional exposure stimulus in PE therapy and as an alternative to exposure solely to the corresponding trauma memory.

## Data Availability

The data that support these findings are available from the corresponding author upon reasonable request and with requisite documentation from Massachusetts General Hospital. Raw data can be requested from the National Institute of Mental Health Data Archive (NDA) using grant number R21 MH128619 at the following link: https://nda.nih.gov/
